# The Involvement of Nitric Oxide in Integration of Plant Physiological and Ultrastructural Adjustments in Response to Arsenic

**DOI:** 10.3389/fpls.2017.00516

**Published:** 2017-04-19

**Authors:** Fernanda S. Farnese, Juraci A. Oliveira, Elder A. S. Paiva, Paulo E. Menezes-Silva, Adinan A. da Silva, Fernanda V. Campos, Cléberson Ribeiro

**Affiliations:** ^1^Laboratório de Ecofisiologia Vegetal, Instituto Federal GoianoRio Verde, Brazil; ^2^Departamento de Biologia Geral, Universidade Federal de ViçosaViçosa, Brazil; ^3^Departamento de Botânica, Instituto de Ciências Biológicas, Universidade Federal de Minas GeraisBelo Horizonte, Brazil

**Keywords:** *Pistia stratiotes*, photosynthesis, programmed cell death, respiration, cell signaling

## Abstract

High arsenic (As) concentrations are toxic to all the living organisms and the cellular response to this metalloid requires the involvement of cell signaling agents, such as nitric oxide (NO). The As toxicity and NO signaling were analyzed in *Pistia stratiotes* leaves. Plants were exposed to four treatments, for 24 h: control; SNP [sodium nitroprusside (NO donor); 0.1 mg L^-1^]; As (1.5 mg L^-1^) and As + SNP (1.5 and 0.1 mg L^-1^, respectively). The absorption of As increased the concentration of reactive oxygen species and triggered changes in the primary metabolism of the plants. While photosynthesis and photorespiration showed sharp decrease, the respiration process increased, probably due to chemical similarity between arsenate and phosphate, which compromised the energy status of the cell. These harmful effects were reflected in the cellular structure of *P. stratiotes*, leading to the disruption of the cells and a possible programmed cell death. The damages were attenuated by NO, which was able to integrate central plant physiological processes, with increases in non-photochemical quenching and respiration rates, while the photorespiration level decreased. The increase in respiratory rates was essential to achieve cellular homeostasis by the generation of carbon skeletons and metabolic energy to support processes involved in responses to stress, as well to maintaining the structure of organelles and prevent cell death. Overall, our results provide an integrated view of plant metabolism in response to As, focusing on the central role of NO as a signaling agent able to change the whole plant physiology.

## Introduction

Arsenic is a toxic metalloid naturally occurring in the environment and that originates primarily from the dissolution of certain types of rocks. High As concentrations, however, are toxic to all the living organisms and occur worldwide, especially as a result of human activities (Gomes et al., 2014; Kumar et al., 2015). Arsenate is the most common form of As in oxygenated environments such as aerobic soils and surface water. As a chemical analog of phosphate, arsenate is easily absorbed by the plant through high-affinity phosphate transporters located in the roots (Castrillo et al., 2013). Once absorbed, the pollutant triggers serious changes in the plant metabolism, affecting both the physiological and the biochemical processes, which may reveal alterations in morphology and in the plant cell organization (de Freitas-Silva et al., 2016).

Much of the damage triggered by As is a result of the increased production of ROS, which can interact with several molecules and modify their structure and function (Alcantara-Martinez et al., 2016). ROS generation naturally occurs as a consequence of aerobic metabolism, and, in normal cell conditions, their homeostasis is carefully balanced, keeping their levels approximately constant. In stressful conditions, however, ROS generation increases quickly and expressively, affecting organelles and plant processes, which, ultimately, interfere in the growth and survival of the plant (Leão et al., 2014; Rofkar et al., 2014).

Plants frequently respond to stressors by reprogramming their physiological processes to ensure the maintenance of homeostasis and the cell functions. Processes such as photosynthesis, photorespiration, and respiration are intrinsically related and essential to the plant’s tolerance to abiotic stresses, being carefully regulated in adverse conditions (Millar et al., 2011; Gupta et al., 2014; Osakabe et al., 2014). This regulation involves both changes in the cellular redox and cell signaling, with the participation of various secondary messengers, such as NO (Millar et al., 2011).

Nitric oxide is a cell signaling molecule particularly important in the plant’s tolerance to stress. In the last few years, several researches showed that NO is able to improve plant tolerance to As (Shukla et al., 2015; Silveira et al., 2015; Andrade et al., 2016). Strong evidences indicate that NO acts as a second messenger, triggering different cellular responses, such as the increase in antioxidant defense systems (Fan et al., 2014). This molecule also acts as a signal in the major physiological process in the plant, promoting, for example, changes in photosynthesis and respiration under several environmental conditions (Farnese et al., 2016). However, most of these studies assess the physiological process in an individual way, without considering that they occur at the same time in the plant cell. As a result, it is still not clear if NO is able to simultaneously alter different physiological processes and therefore reprogram the plant metabolism in order to increase plant tolerance to As. In addition, few studies focus the response of plants to heavy metals and the effect of NO in cellular organelles. In this way, there are few data that enforces the complementarity between structure and function, which limits the comprehension of tolerance mechanisms. Seeking to fill this gap, the aim of this study was to evaluate the effects of As and NO on the physiology, morphology and ultrastructure of *Pistia stratiotes*, an aquatic macrophyte able to absorb and accumulate large amounts of As (Farnese et al., 2013) and recommended for remediation of surface waters (Lu et al., 2011). We hypothesized that NO would be able to improve the plant tolerance to As by reprogramming the physiological process, which would result in the attenuation of the cellular damages caused by the pollutant.

## Materials and Methods

### Plant Material

*Pistia stratiotes L.* plants (Araceae) were collected in non-polluted dams in a horto forest at the Federal University of Viçosa, Viçosa, Minas Gerais State, Brazil (20°45′25.0″ S 42°52′25.5″ W), with average ambient temperature between 20.6 and 25.2°C and average annual precipitation of 1229 mm. The plants were sterilized with sodium hypochlorite (1%) for 1 min, transferred to Clark’s (1975) nutrient solution (14 of ionic strength, pH 6.5) and maintained in a plant growth room with controlled temperature and light (25 ± 2°C, 230 μmol m^-2^ s^-1^) and a photoperiod of 16 h. The plants remained in these conditions for 3 days for acclimation. After the acclimation period, the plants were transferred to containers containing 0.5 L of Clark’s (1975) nutrient solution, 14 of ionic strength, pH 6.5, and exposed to four treatments: control (nutrient solution only); SNP (0.1 mg L^-1^); As (1.5 mg L^-1^) and As + SNP (1.5 and 0.1 mg L^-1^, respectively). Arsenic was supplied as sodium arsenate dibasic heptahydrate (Na_2_HAsO_4_⋅7H_2_O). SNP is a substance widely used in biochemical studies as a NO donor. The concentration of As chosen was the maximum concentration at which the plant still showed positive growth rate (in higher concentrations the growth rate was negative due the loss of root system) and the concentration of SNP selected was one in which the index of tolerance to As was approximately 50% (Farnese et al., 2013). The plants remained in these conditions for 24 h.

### Determination of Arsenic Concentration

In order to determine the concentration of As absorbed, all the young fully expanded leaves were collected, washed in distilled water and kept in a conventional oven at 80°C until a constant dry weight was obtained. The dried material was macerated and digested in a mixture of nitric and perchloric acid (Marin et al., 1993), and the concentration of As was determined through inductively coupled plasma emission (Optima 3300 DV, Perkin-Elmer, Norwalk, CT, USA).

### Nitric Oxide Measurement

For fluorometric NO determination, the cell-permeable diacetate (DAF-2DA) was used (Alexis Biochemicals, Gruenberg, Germany). Leaf fragments were sectioned and incubated with 15 μM DAF-2DA in the dark for 20 min at ambient temperature. Fluorescent signals were detected using a confocal microscope (Zeiss LSM 510). Emission light was collected at 515 ± 10 nm, and excitation was at 488 ± 10 nm. Signal intensities were quantified using Photoshop (Adobe Systems). Fluorescence was expressed as percentage of the control levels (Hai et al., 2006).

### Determination of the Concentration of Reactive Oxygen Species

For determining the concentration of superoxide anion ($O_{2}^{•–}$), samples of 50 mg of leaves were incubated in an extraction medium consisting of 100 μM disodium salt of ethylenediaminetetra-acetic acid (EDTA), 20 μM NADH, and 20 mM sodium phosphate buffer, pH 7.8 (Mohammadi and Karr, 2001). The reaction was initiated by introducing 100 μL of 25.2 mM epinephrine in HCl 0.1 N. Samples were incubated at 28°C with stirring for 5 min. The absorbance reading was taken at 480 nm for 5 min (Boveris et al., 2002).

The concentration of hydrogen peroxide (H_2_O_2_) was measured using 200 mg of leaf samples homogenized in extraction medium and centrifuged at 10,000 × *g* for 15 min at 4°C (Kuo and Kao, 2003). Aliquots of 50 uL of supernatant were added to the reaction medium containing 100 μM FeNH_4_SO_4_, 25 mM sulfuric acid, 250 μM xylenol orange and 100 mM sorbitol (Gay and Gebicki, 2000). Samples were kept in the dark for 30 min, and the absorbance was determined at 560 nm. H_2_O_2_ concentrations were estimated based on a calibration curve.

### Photosynthetic Pigments, Chlorophyll Fluorescence, and Gas Exchange

To determine the concentration of chlorophyll *a* and chlorophyll *b*, two leaf disks of 0.5 cm diameter were collected, weighed and incubated in 5 mL of DMSO (dimethylsulfoxide), saturated with calcium carbonate, for 24 h (Wellburn, 1994). After this time, the absorbance reading was performed in a spectrophotometer at wavelengths of 665 and 649 nm to determine the concentration of chlorophyll *a* and *b*, respectively.

Leaf gas exchange and chlorophyll *a* fluorescence were measured simultaneously with an open-flow infrared gas exchange analyzer system, equipped with a leaf chamber fluorometer (LI-6400XT, Li-Cor, Lincoln, NE, USA). Light-saturated net CO_2_ assimilation rates (*A*), stomatal conductance (*g_s_*) and internal CO_2_ concentration (*C_i_*) were determined on attached, fully expanded leaves, under saturating light at leaf level (1000 μmol photon m^-2^ s^-1^) and 400 μmol CO_2_ mol^-1^ air. Analysis of the minimal fluorescence (*F*_0_) was performed before the dawn period via the excitation of the leaf tissues with modulated red light of low intensity (0.03 μmol photon m^-2^ s^-1^). To obtain maximum fluorescence (*F*_m_), saturation pulses of approximately 8.000 μmol photon m^-2^ s^-1^ were applied for 0.8 s. The variable fluorescence (*F*_v_) was determined by the difference between *F*_0_ and *F*_m_, and, from these values, the potential quantum yield of the photosystem II was calculated (*F*_v_/*F*_m_) (van Kooten and Snel, 1990). At mid-morning, the same leaves measured at predawn were acclimated to actinic light (1.000 μmol photon m^-2^ s^-1^) for 60 s in order to obtain the transient fluorescence (*F*_s_), followed by a saturating light pulse to estimate the maximal fluorescence (*F*_m_′). Finally, the actinic light was switched off, and far-red illumination was applied (2 μmol photon m^-2^ s^-1^) to measure the light-adapted initial fluorescence (F0′). With these parameters, the actual PSII photochemical efficiency (Φ_PSII_), the quantum yield of CO_2_ assimilation (Φ_CO2_) and the non-photochemical quenching (NPQ) were estimated as proposed by Maxwell and Johnson (2000).

### Mitochondrial Respiration

The rate of mitochondrial respiration in the darkness (*R*_dark_) was measured before dawn using the infrared-gas analyzer mentioned above, and it was divided by two (R_dark_/2) as a proxy for estimation of the respiration in the light [*R*_light_ (rate of non-photorespiratory mitochondrial CO_2_ release)], according to Martins et al. (2013).

### Photorespiratory Rates and Intermediates of Photorespiration

The photorespiration rate (P_R_) was estimated using the combined data of gas exchange and chlorophyll *a* fluorescence described above. The flow of electrons used for the carboxylation (ETRc) and oxygenation (ETRo) by Rubisco (ribulose-1,5-bisphosphate carboxylase/oxygenase) was calculated (Epron and Dreyer, 1993; Valentini et al., 1995).

To determine the concentration of intermediates of photorespiration (serine, glycine, and glycerate), lyophilized leaf samples were subjected to methanolic extraction, and the compounds were quantified by a gas chromatography coupled with mass spectrometry (GC–MS) as described by Lisec et al. (2006). The GC–MS system used was composed of autosampler CTC CombiPAL, gas chromatographer (Agilent 6890N) and mass spectrometry (Leco Pegasus III TOF-MS, operated in positive ionization mode). The chromatogram and mass spectrum were evaluated using the program TAGFINDER (Luedemann et al., 2008). The compounds were identified by comparison with authentic standards (Kopka et al., 2005).

### Determination of Carbohydrates

Lyophilized leaf samples were subjected to an ethanolic extraction and, in the soluble fraction, the levels of glucose and sucrose were determined; whereas, in the ethanol-insoluble pellet, the levels of starch were determined, according to the methodology proposed by Fernie et al. (2001).

### Micromorphologic and Ultrastructural Alterations

To analyze the foliar micromorphology, samples of the middle region of the leaves were collected and fixed in 2.5% glutaraldehyde in 0.1 M sodium phosphate buffer, pH 7.2, for 2 h. After this period, the samples were dehydrated in an ascending ethanol series, subjected to drying to the critical point with liquid CO_2_ (Balzers Model PCD 020, Bal-Tec, Liechtenstein), fixed in stubs and finally subjected to metal deposition with gold in a Balzers equipment (FDU Model 010, Bal-Tec, Balzer, Liechtenstein). Photographic observations and documentation were obtained in a LEO scanning electron microscope (Model 1430VP).

For visualization of ultrastructural damages, *P. stratiotes* leaf samples were fixed in 2.5% glutaraldehyde in 0.1 M sodium phosphate buffer, pH 7.1, for 4 h. After that, the samples were post-fixed in 1% osmium tetroxide and dehydrated in an ethanol series. The material was then included in a Spurr resin, and ultra-thin sections with 50 nm were obtained using an ultramicrotome (Model UCT, Leica Microsystems, Inc., Deerfield, IL, USA), equipped with a diamond knife. Sections were collected on the copper grids and contrasted with uranyl acetate and lead citrate (Reynolds, 1963). The samples were observed with a transmission electron microscope Model Tecnai G2-Spirit (Philips/FEI Company, Eindhoven, Netherlands) coupled with a digital camera.

### Statistical Analyses

The experiments were carried out in a completely randomized design, with five repetitions; the data were submitted to ANOVA, and the means were compared using the SNK test (Student Newman Keuls), at 5% probability. Principal component analysis (PCA) was done to obtain a more accurate grouping of the samples and to determine the most discriminant spots. All statistical analyzes were performed using the statistical software SAS 9.1 (SAS Institute, Inc. 2004, Cary, NC, USA).

## Results

### Arsenic Absorption and ROS Generation

Plants of *P. stratiotes* exposed to As were able to absorb, translocate, and accumulate large amounts of the pollutant in the leaves, which was followed by an increase in ROS generation (**Table 1**). Although the presence of SNP did not alter the pattern of As accumulation compared with the treatment containing only the metalloid, the pattern of ROS generation was modified. We observed that the addition of NO kept the O_2_^•–^ and H_2_O_2_ concentrations in levels similar to the control.

**Table 1:** As concentration, reactive oxygen species production and DAF fluorescence in *P. stratiotes* plants exposed to arsenic alone or in combination with SNP.

*Treatments*	As concentration (mg g^-1^ DW)	H_2_O_2_ (μmol g^-1^ FW)	$O_{2}^{•–}$ (nmol g^-1^ FW)	Fluorescence (% of control)
Control	0 b	2.14 b	23.21 b	100 c
SNP	0 b	1.85 b	19.35 b	132 b
As	0.589 a	3.36 a	42.51 a	126 b
As+SNP	0.565 a	2.01 b	27.03 b	158 a

*Means followed by the same letter were not significantly different according to SNQ test at 5% probability.*

### Nitric Oxide Concentration

**FIGURE 1:**
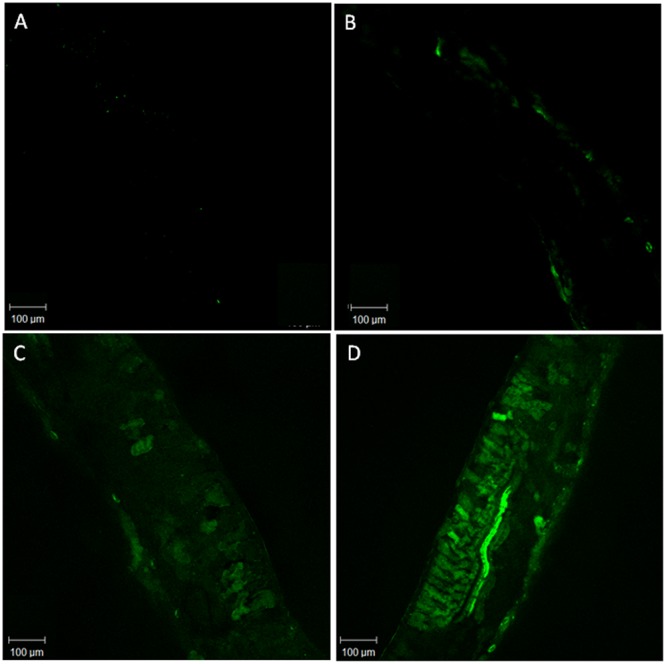
**Representative images illustrating the confocal laser immunofluorescent detection of NO in *Pistia stratiotes* leaves.** NO was detected by its bright green fluorescence after incubation with DAF-2DA. Negative control without DAF-2DA **(A)**; Control plants with DAF-2DA **(B)**; Plants exposed to arsenic and incubated with DAF-2DA **(C)**; Plants exposed to arsenic + SNP and incubated with DAF-2DA **(D)**.

The DAF fluorescence, used as a parameter to determine NO concentration, was higher in the treatments SNP and As+SNP indicating that NO released by SNP solution was efficiently absorbed by the plants (**Table 1**). Plants exposed only to As also had greater fluorescence than the control, showing that *P. stratiotes* is able to produce NO in stress conditions (**Figure 1**).

### Effects of Arsenic and SNP on Plant Physiological Processes

#### Photosynthesis

**FIGURE 2:**
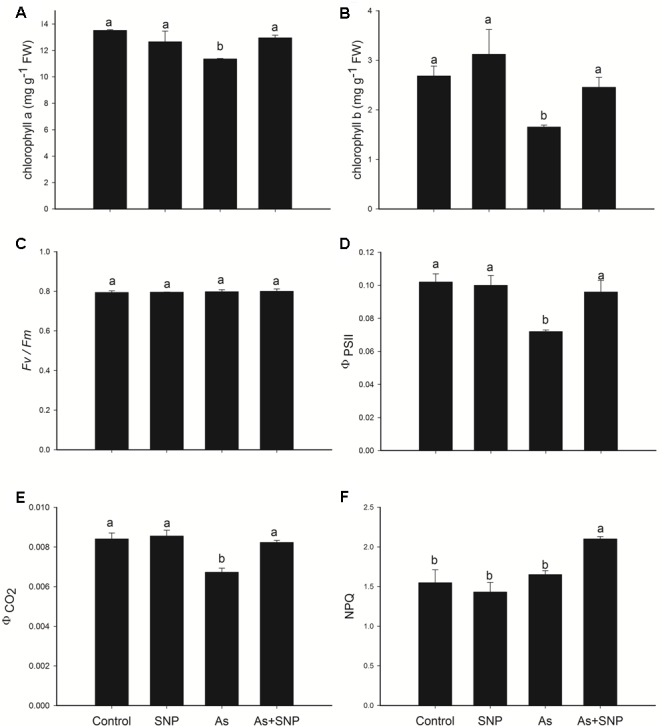
**Variation in the leaf chlorophyll *a* fluorescence parameters in *P. stratiotes* exposed to As and SNP during 24 h.** The parameters included chlorophyll *a* content **(A)**; chlorophyll *b* content **(B)**; maximal photochemical efficiency of PSII (*F*_v_/*F*_m_) **(C)**; the actual PSII photochemical efficiency (Φ_PSII_) **(D)**; the quantum yield of CO_2_ assimilation (Φ_CO2_) **(E)**; and the non-photochemical quenching (NPQ) **(F)**. Means followed by the same letter were not significantly different according to SNK test at 5% probability.

The exposure of *P. stratiotes* to As triggered changes in all physiological processes analyzed. Regarding photosynthesis, a central process in plant metabolism, several parameters were affected by the metalloid. The concentrations of chlorophyll *a* and *b* significantly decreased (**Figures 2A,B**), especially for chlorophyll *b*. Changes in photosynthetic pigments reflected on the photochemical process of photosynthesis and, although the pollutant has not triggered change in the potential quantum yield (*F*_v_/*F*_m_) (**Figure 2C**), the actual PSII photochemical efficiency (Φ_PSII_) and the quantum yield of CO_2_ assimilation (Φ_CO2_) were affected (**Figures 2D,E**). The addition of SNP in to the solution containing As was, however, able to attenuate the toxic effect of the metalloid on chloroplastid pigments, as well as Φ_PSII_ and Φ_CO2_. In the AS + SNP treatment, we also observed an increase in the NPQ (**Figure 2F**).

**FIGURE 3:**
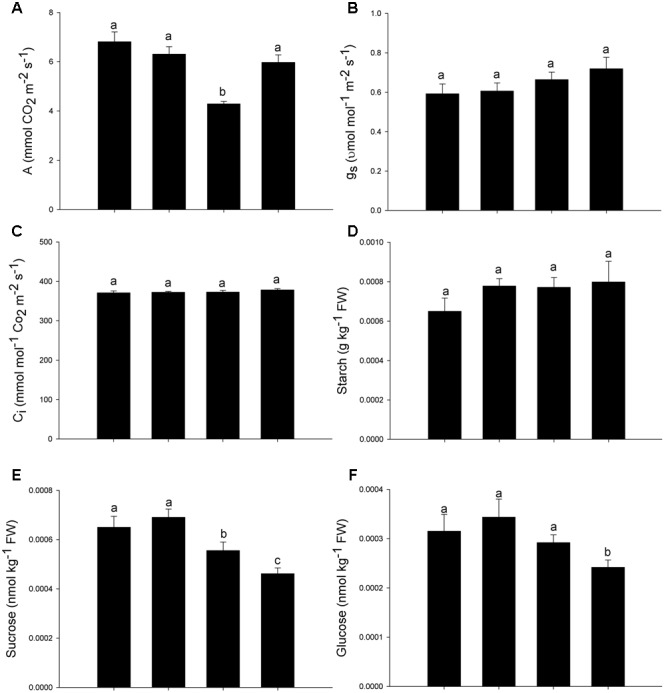
**Variation in the leaf gas exchange parameters in *P. stratiotes* exposed to As and SNP during 24 h.** The parameters included net photosynthesis rate (*A*) **(A)**, stomatal conductance to water vapor (*g_s_*) **(B)**, internal CO_2_ concentration (*C_i_*) **(C)**, starch concentration **(D)**, sucrose concentration **(E)**, and glucose concentration **(F)**. Means followed by the same letter were not significantly different according to SNK test at 5% probability.

Besides chlorophyll fluorescence, the gas exchange traits were also affected by As, with decreases in net CO_2_ assimilation rates (*A*) (**Figure 3A**), while the stomatal conductance (*g_s_*) and the internal CO_2_ concentration (*C_i_*) remained constant (**Figures 3B,C**). The addition of SNP was able to maintain the carbon assimilation rate in levels similar to those observed in control plants (**Figure 3A**). Although the starch concentration remained constant, the concentration of sucrose decreased in all treatments with As. The decrease in sucrose concentration was more expressive when As was supplied in combination with the SNP (**Figures 3D,E**). The glucose concentration was affected only in the treatment As+SNP (**Figure 3F**).

#### Respiration and Photorespiration

**FIGURE 4:**
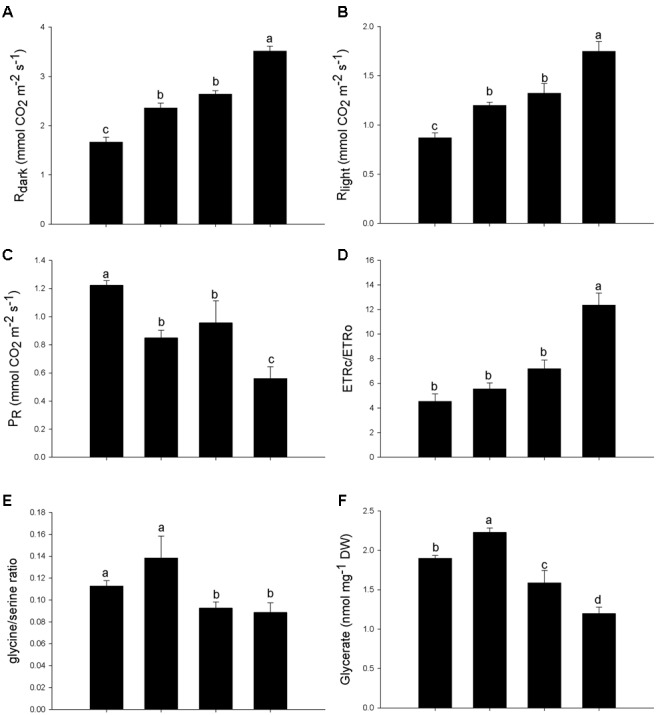
**Variation in the respiration and photorespiration parameters in *P. stratiotes* exposed to As and SNP during 24 h.** The parameters included rate of mitochondrial respiration in the darkness (R_dark_) **(A)**; rate of non-photorespiratory mitochondrial CO_2_ release (R_light_) **(B)**; photorespiratory rate (P_R_) **(C)**; ratio between flow of electrons used for the carboxylation (ETRc) and oxygenation (ETRo) (ETRc/ETRo) **(D)**; glycine/serine ratio **(E)**; glycerate concentration **(F)**. Means followed by the same letter were not significantly different according to SNK test at 5% probability.

Both Arsenic and SNP increased nocturnal respiration (R_dark_) and mitochondrial respiration (R_light_) in leaves of *P. stratiotes*, however, the increase was more significant when SNP and As were supplied in combination (**Figures 4A,B**). The photorespiratory rate (P_R_) of *P. stratiotes* decreased in plants exposed both to As and SNP and, once again, these decreases were more pronounced when the two substances were supplied together (**Figure 4C**). Although the carboxylation rate of Rubisco (ETRc) has been changed only in the treatment containing the metalloid, the oxygenation rate (ETRo) decreased in the treatments containing both As and SNP. As a result, the ETRc/ETRo ratio increased only in plants exposed simultaneously to As and SNP (**Figure 4D**). Accordingly with P_R_ reduction, the glycine/serine ratio and the concentration of glycerate also decreased in response to SNP and As (**Figures 4E,F**).

#### Micromorphologic and Ultrastructural Alterations

**FIGURE 5:**
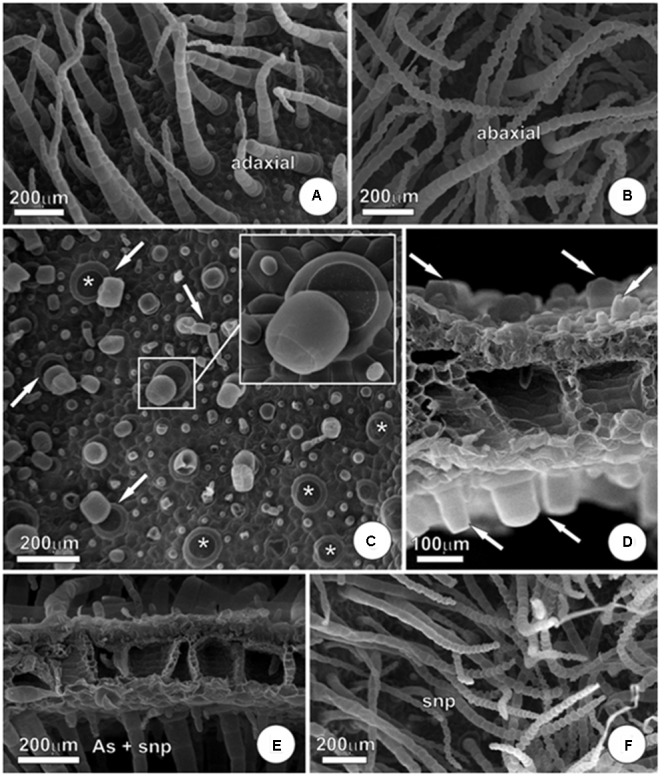
**Micromorphology of *P. stratiotes* leaves showing effects of As and SNP. (A,B)** Control plants. Note the entire and well-preserved trichomes in both adaxial **(A)** and abaxial **(B)** leaf surface. **(C)** Adaxial leaf surface of plant exposed to As during 24 h. Note the trichome absence. Arrows indicate the last step of abscission with only the trichome base remaining (^∗^) and, in the insert, a detail of trichome disassemble. **(D)** Sectioned leaf showing severe trichome abscission in plant exposed to As. Arrows indicate trichome basal cell in both surfaces. **(E,F)** Leaf from plants exposed to As + SNP. In **(E)**, we can observe trichomes in both adaxial and abaxial leaf surfaces and, in **(F)**, a detail of abaxial leaf surface, similar to the one observed in control plants.

Exposure to arsenic has triggered several changes on leaf micromorphology and ultrastructure of parenchyma cells in *P*. *stratiotes* leaves. Concerning the micromorphology, the main change triggered by the pollutant was in the amount of trichomes in the epidermis of leaves. In plants that remained in the solution without As (**Figures 5A,B**), it was possible to visualize substantial amount of non-glandular trichomes in both adaxial and abaxial faces of the leaves. However, exposure to As had a harmful effect on trichomes, substantially reducing the amount of these epidermal appendages.

The leaf trichomes of *P*. *stratiotes* are simple, uniseriate and multicellular, formed by a large basal cell and an axis composed of up to 30 cells with a diameter that shows a reduction in the acropetal direction, culminating with tapered apical cell (**Figures 5A,B**). In plants exposed to As, we observed abscission of cells that compose the axis of trichomes, remaining in many cases only the basal cell (**Figures 5C–E**). The process of abscission here observed in trichomes takes place by rupture of the middle lamella in order to maintain the integrity of other portions of the cell walls, while preserving the integrity of the cells (**Figures 5C,D**). The addition of SNP was able to partially alleviate this damage and, despite the fact that it was still possible to view trichomes collapsed and trichome abscission in the As + SNP treatment, the global appearance of the epidermis was very similar to that observed in control plants (**Figures 5E,F**).

**FIGURE 6:**
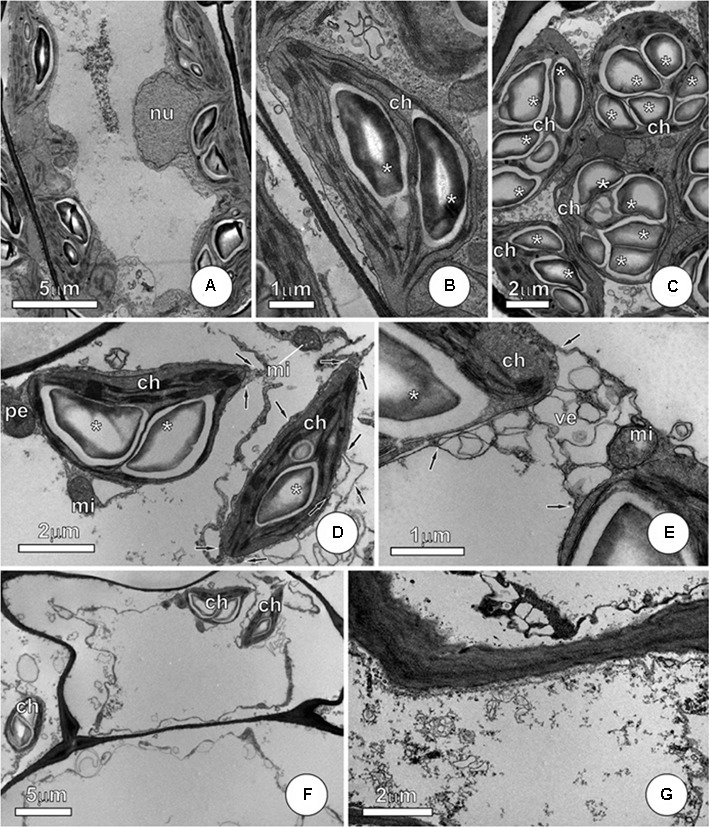
**Ultrastructure of mesophyll cells from *P. stratiotes* leaves showing effects of As. (A,B)** Control plants showing integrity of protoplast, with well-preserved membrane systems and normal chloroplasts with starch grains (^∗^). **(C–G)** Mesophyll cells from plants exposed to As. **(C)** Cell showing the initial stage of As damage. Notice the well-preserved organelles and a discrete vesiculation in ground cytoplasm (arrows). **(D,E)** Chloroplast showing outer membrane alterations (arrows). Note intense vesiculation but well-preserved inner membranes and starch grains (^∗^). **(F,G)** General view **(F)** and detail of severe damaged cells. Note that chloroplasts and few mitochondria persist, but plasma membrane collapse causes cytoplasm leaking, with only collapsed residues remaining (ch, chloroplast; mi, mitochondria; nu, nucleus; ve, vesicle).

**FIGURE 7:**
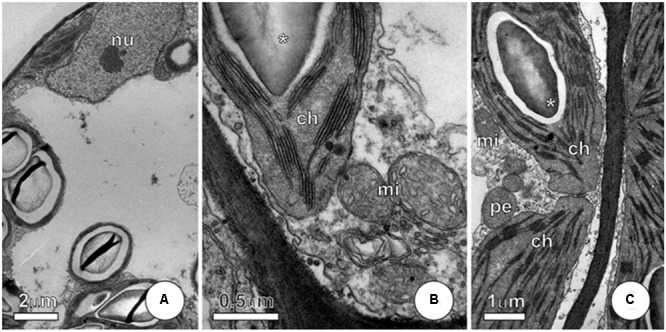
**Ultrastructure of mesophyll cells from *P. stratiotes* leaves showing effects of As + SNP. (A–C)** Cells showing integrity of protoplast, with well-preserved protoplast and no evidence of membrane alterations. Note that chloroplast shows starch grains (^∗^) and, like mitochondria, maintains normal structure (ch, chloroplast; mi, mitochondria; nu, nucleus; pe, peroxisome).

In *P*. *stratiotes*, the mesophyll cells showed protoplast affected by exposure to As, with the most conspicuous changes observed in the membrane system, with particular effect on the plasma membrane, vacuole, chloroplasts, and mitochondria (**Figure 6**). Plants that were not exposed to As show integrity of protoplast, with well-preserved membrane systems and normal chloroplasts (**Figures 6A,B**). In mesophyll cells, we noticed that damage to the membrane system resulted in collapse of the protoplast in plants (**Figures 6C–E**), with sharp contrast between these arsenic-exposed cells and those of the control group (**Figures 6A,B**) or of the treatment As + SNP (**Figures 7A–C**). The changes observed in mesophyll cells subjected to As were evident, despite the fact that different degrees of cell disorder were observed, including the presence of cells apparently healthy, just showing discrete vesiculation in ground cytoplasm (**Figure 6C**). An intense vesiculation was observed in all membrane systems, which causes plasma membrane ruptures and ground cytoplasm extravasation (**Figure 6D**). Among organelles, chloroplasts detach by intense structural alterations, showing recurrent vesiculation of the outer membrane (**Figure 6D**), as well as expansion in the thylakoid membranes. Breaking traces of the mitochondrial membrane (**Figure 6E**) were also observed, although mitochondria and chloroplasts have shown integrity even in cases of severe damages to cells. In necrotic areas, the protoplast was fully collapsed, and, progressively, just protoplast remnants could be seen (**Figures 6F,G**).

Plants subjected to As + SNP hardly have visual changes and, in some cases, we observed only discrete chlorosis. Mesophyll cells showed protoplast integrity without evidences of membrane alterations (**Figures 7A–C**). Chloroplast and mitochondria showed normal structure (**Figures 7B,C**), like those from control plants.

### Multivariate Statistical Analysis

**FIGURE 8:**
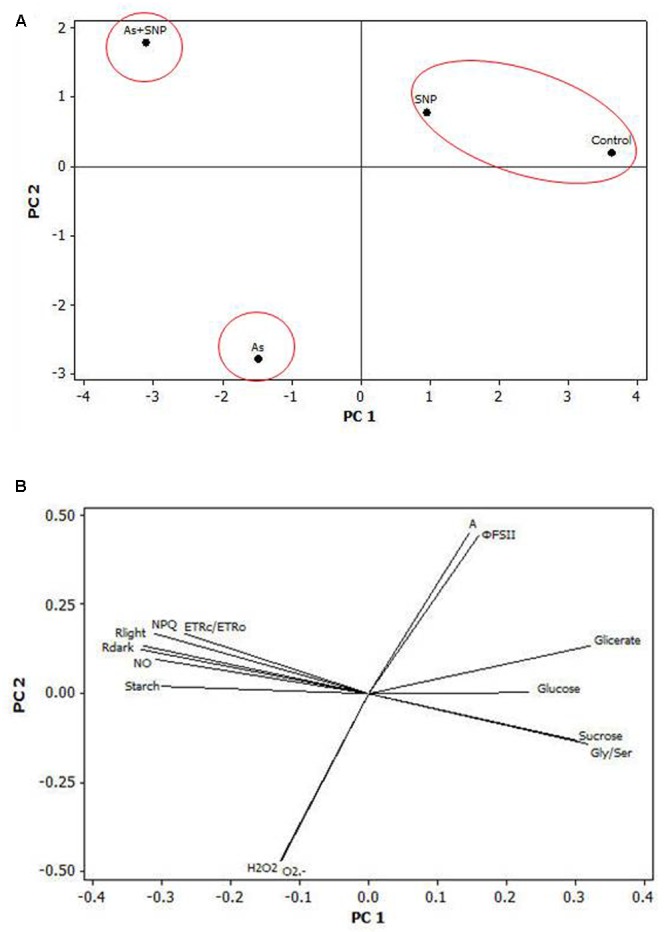
**Multivariate analysis (PCA analysis).** Two-dimensional PCA biplots showing associations between experimental groups and analysis spots generated by principal component analysis (PCA). The segregation of the experimental groups **(A)** and the correlation coefficients for all the analysis **(B)** were plotted in the first two component spaces.

The PCA analysis has shown the most discriminant spots between the treatments. Three main clusters were obtained (**Figure 8A**). This clustering indicated that the treatments Control and SNP had similar features, while As and As+SPN treatments had a distinct separation. The main variables responsible for the separation between the As and As+SNP treatments were the accumulation of NO and changes in respiratory parameters (**Figure 8B**).

## Discussion

The absorption and accumulation of As in the leaves of *P. stratiotes* interfered in physiology, ultrastructure and micromorphology of plants. In fact, As is able to affect several aspects of the cellular functioning in plants, as observed in *Azolla caroliniana* (Rofkar et al., 2014), *Lemna gibba* (Leão et al., 2013) and *Eichhornia crassipes* (Andrade et al., 2016). These harmful effects are due, mainly, to the increase in the generation of ROS, such as superoxide anion and hydrogen peroxide (Srivastava et al., 2013; Mishra et al., 2014). The addition of SNP, in turn, decreased the production of ROS and, therefore, the damages triggered by the metalloid were also reduced, as has been observed for several other plants exposed to As (Farnese et al., 2013; Fan et al., 2014; Saeid et al., 2014). Although the reduction of ROS due to the addition of SNP is typically associated only with the enhancement of antioxidant defense systems (Jin et al., 2010; Saeid et al., 2014), in the case of *P*. *stratiotes*, this reduction involved the coordination of central physiological processes of the primary metabolism.

Since chloroplasts are one of the main sites of ROS production in cells, they are commonly the more sensitive organelles to the damages triggered by As. In *P. stratiotes*, severe structural damages to internal membranes of chloroplasts were observed, as well as in leaves of *Leucaena leucocephala* (Schneider et al., 2013) and *Pteris vittata* (Li et al., 2006) exposed to As. In the outer membrane of chloroplasts, in turn, we observed the occurrence of vesiculation, indicating the beginning of the degradation process of these organelles, a common response to different stresses (Wang and Blumwald, 2014). Not only the degradation of chloroplasts but also changes in internal membranes of this organelle have a strong impact on photosynthesis and triggered from the reduction in the concentration of photosynthetic pigments to the decrease in the rates of carbon assimilation (Schneider et al., 2013). In fact, exposure to As was able to affect the photosynthesis in the leaves of *P*. *stratiotes* at different levels, indicating that the decrease in photosynthesis rates was mainly due to the disruption and degradation of chloroplasts.

In addition to changes in chloroplasts, reductions in the concentrations of chlorophyll *a* and *b* may also have been caused by other factors such as increased activity of chlorophyllase and reduction of chlorophyll synthesis. The decrease in the concentration of photosynthetic pigments is frequently associated with the exposure to As (Srivastava et al., 2013) and has already been observed in other aquatic plants exposed to this metalloid, as *Lemna minor* and *Azolla caroliniana* (Rofkar et al., 2014). The depletion in photosynthetic pigments content was probably one of the reasons for decrease in the efficiency of PSII (Baker, 2008) since As did not damage proteins and components of PSII, as evidenced by the maintenance of constant values of *F*_v_/*F*_m_. The addition of SNP was able to maintain the structure of the chloroplasts and the chlorophyll levels and, therefore, the fluorescence parameters were restored to the normal. Another factor that possibly contributed to the maintenance of Φ_PSII_ in the AS + SNP treatment was the increase in NPQ, an indicator of thermal dissipation in PSII antenna (Baker, 2008). The increase in NPQ is commonly observed in plants exposed to stressors and represents an important adaptive mechanism (Carbonera et al., 2012).

The decrease triggered by As in Φ_CO2_ as well as in net CO_2_ assimilation rates is a reflection of the changes in chloroplasts and the consequent reduction in PSII efficiency, since changes were not observed in *g_s_* and *C_i_* (Xing et al., 2013). The reduction in fixation of CO_2_ may have occurred also because of the decrease in gene expression of Rubisco and other enzymes of the Calvin cycle in response to the pollutant (Finnegan and Chen, 2012). However, the photosynthetic responses of plants to As appears to be variable among the species. Indeed, while in some plants the photosynthetic limitations triggered by As are essentially biochemical (Srivastava et al., 2013), as observed for *P. stratiotes*, in *Oryza sativa* the decrease in the net carbon assimilation after exposure to the metalloid was mainly a consequence of the stomatal closure (Sanglard et al., 2014). In the current study, the impairment of the photosynthetic process induced by As was corroborated by the low concentration of sugars in the plant cells. It is interesting to note, however, that the addition of SNP promoted further reductions in the concentration of glucose and sucrose, which is not associated with a reduction on photosynthetic rate, since the carbon fixation process has been restored. It is therefore likely that the sharp decrease in the concentration of sugars in plants exposed to As + SNP is associated with the high respiratory rates.

Arsenic, provided separately, promoted increases in plant respiration. This result is in sharp contrast with transcriptional analyses in *Arabidopsis thaliana* exposed to As which indicated a decrease in the enzyme activity of the citric acid cycle (Abercrombie et al., 2008), but the respiratory activity of the plants was not measured. In *P. stratiotes* exposed to the metalloid the increment in respiratory rates were not accompanied by the restoration of cellular homeostasis. In other words, the increase in respiration did not contribute to the restoring of photosynthesis rates, to decrease the ROS concentration or to the maintenance of cell structure. The increased respiration in plants exposed to As is probably a result of the chemical similarity between the phosphate and arsenate, which compete for the same active site of the mitochondrial ATP synthase (Moore et al., 1983). This competition results in the synthesis of a highly unstable product As-ADP, causing a decrease in ATP concentration. Low ATP levels are a signal that triggers the increment in respiratory activity, which leads to the generation of more ADP-As. Therefore, although increases in respiration are important in stress conditions to provide carbon skeletons (Dutilleul et al., 2003), the occurrence of futile cycles of As-ADP generation compromises the energy status of the cell and can contribute to the increase of ROS production (Finnegan and Chen, 2012). The alterations in cell respiration of plants subjected only to As probably would not hold for longer periods of exposure, as the metalloid triggered the disruption of mitochondrial membrane, which would compromise the respiratory process.

Besides As, NO also improved the respiration rates in plant cells. Unlike what occurs for As, however, the change mediated by NO in the respiration rates was important to achieve cellular homeostasis as indicated by the PCA analysis. Indeed, increases in respiratory process contribute to the plant tolerance by the generation of carbon skeletons and metabolic energy to support processes involved in responses to stress (Fernie et al., 2004; Bolton, 2009). In addition, the improve of respiration rates may have also contributed to maintaining the structure of organelles, once the adequate energy status of the cell is necessary for the synthesis and restoration of cell membranes (Liu et al., 2016) and to decreased ROS generation (Millar et al., 2011). The mechanisms by which NO mediates the regulation of mitochondrial respiration to protect the cells are not fully understood, but evidence indicates the involvement of multiple pathways, including the increase of the pathway of cytochrome c oxidase and the route of alternative oxidase (Jhanji et al., 2012; Shan et al., 2012).

Unlike what was observed in respiration, *P*. *stratiotes* treated with As showed decreases in the photorespiration, and this reduction was more expressive when the pollutant was supplied together with SNP. In fact, As+SNP decreased the oxygenation rate of Rubisco, increased the ETR_C_/ETR_O_ ratio, decreased the concentration of glycerate and decreased the glycine/serine ratio, one of the main biochemical markers for photorespiration rate (Novitskaya et al., 2002). All of these parameters indicate a reduction of photorespiration, which could be the consequence of the inactivation of enzymes related to photorespiratory process. Evidences of the inhibitory role of NO on photorespiration have also been observed in *Arabidopsis* (Corpas and Barroso, 2016). Several enzymes involved in photorespiration are targets of S-nitrosylation by NO, as the enzyme glycolate oxidase (GOX), which is inhibited by this process. The enzyme GOX is a key enzyme in photorespiration and its activity results in the formation of H_2_O_2_. Thus, it is believed that NO might be important in the regulation of the levels of ROS by post-translational modifications of one of the major enzymes responsible for H_2_O_2_ production (Ortega-Galisteo et al., 2012). Such regulation is necessary because, although the photorespiration plays important physiological roles, under stress conditions this process can be responsible for the generation of up to 70% of all cellular H_2_O_2_ and is considered the main source of ROS in plants submitted to As (Gupta et al., 2013).

In addition to the changes in physiological processes in chloroplasts and mitochondria, it should also be considered that the presence of As disrupted all the protoplast of the mesophyll cells of *P*. *stratiotes.* It is likely that the excess of ROS from plants exposed to As are involved in the disruption of the membrane system, especially in plasma and vacuolar membranes. In the case of the vacuolar membrane, it has been suggested that ROS participate in the cell signaling cascade that culminates in the activation of the vacuolar enzyme of processing, which modifies the structure and causes collapse of the membrane of the vacuole (Li et al., 2013). Additionally, it is likely that the increase in ROS concentration has a direct effect on the denaturation of the cytosol through the oxidation of biomolecules (Jin et al., 2010). In relation to trichomes, the premature senescence was also observed in *Ocimum basilicum* exposed to As and probably is related to the preferential accumulation of the pollutant in these structures (Biwas et al., 2015).

The maintenance of the integrity of some organelles, especially mitochondria, until the cell death, is an evidence of the occurrence of programmed cell death in *P*. *stratiotes* exposed to As (Schussler and Longstreth, 1996). In this process, the increase in ROS concentration acts as a signal that ultimately changes the pattern of gene expression and triggers cell death (Laloi et al., 2006). In plants subjected to As, the coexistence of cells with distinct levels of structural damage, including cells with normal appearance, proved to be compatible with the symptoms perceived externally on the leaves. Exposure to As leads to necrotic patches of circular outline, indicating that cell death cases occurred at specific regions and seemed to progress to cause death of the entire leaf. The ultrastructural analysis showed that this necrosis was a consequence of membrane vesiculation, obviously causing rupture and cytoplasm leakage. Evidence for programmed cell death was not observed in the As + SNP treatment. It is believed that NO is able to retard or prevent this process, which is probably a result of its influence on the concentration of ROS (Beligni et al., 2002).

Based on the observed results, it is possible to conclude that NO triggered signaling cascades that altered physiological processes of the plants, particularly in respiration and photorespiration, so as to enable the maintenance of net CO_2_ assimilation and minimize the generation of ROS, which consequently reduced the damage in cell organelles, as well as in micromorphology. Thus, NO has a fundamental role in the adaptation and integration of physiological processes of *P*. *stratiotes* under stress conditions triggered by As, acting as a global mediator and being crucial to the maintenance of cellular homeostasis and tolerance of the plant to the pollutant.

## Author Contributions

FF designed all the experiments, measured the gas exchange, the chlorophyll fluorescence, the chloroplastic pigments, the concentration of ROS and NO measurement. She also made data analysis and manuscript preparation. PM-S assisted in the measurement of the physiological parameters and determined the concentration of photorespiratory intermediates and carbohydrates. FC, AS, and CR measured arsenic concentration and micromorphologic alterations. EP analyzed ultrastructural alterations. JO and EP reviewed the study as an adviser and a co-adviser, respectively.

## Conflict of Interest Statement

The authors declare that the research was conducted in the absence of any commercial or financial relationships that could be construed as a potential conflict of interest.

## Abbreviations

As: arsenic
NO: nitric oxide
ROS: reactive oxygen species
SNP: sodium nitroprusside
