# Corrigendum: The Involvement of Nitric Oxide in Integration of Plant Physiological and Ultrastructural Adjustments in Response to Arsenic

**DOI:** 10.3389/fpls.2017.00979

**Published:** 2017-06-02

**Authors:** Fernanda S. Farnese, Juraci A. Oliveira, Elder A. S. Paiva, Paulo E. Menezes-Silva, Adinan A. da Silva, Fernanda V. Campos, Cléberson Ribeiro

**Affiliations:** ^1^Laboratório de Ecofisiologia Vegetal, Instituto Federal GoianoRio Verde, Brazil; ^2^Departamento de Biologia Geral, Universidade Federal de ViçosaViçosa, Brazil; ^3^Departamento de Botânica, Instituto de Ciências Biológicas, Universidade Federal de Minas GeraisBelo Horizonte, Brazil

**Keywords:** *Pistia stratiotes*, photosynthesis, programmed cell death, respiration, cell signaling

In the original article, there was a mistake in the legend for Figure 1 as published. Figure 1 will be replaced and therefore the caption should also be changed. The correct legend appears below. The authors apologize for this error and state that this does not change the scientific conclusions of the article in any way.

**Figure 1:**
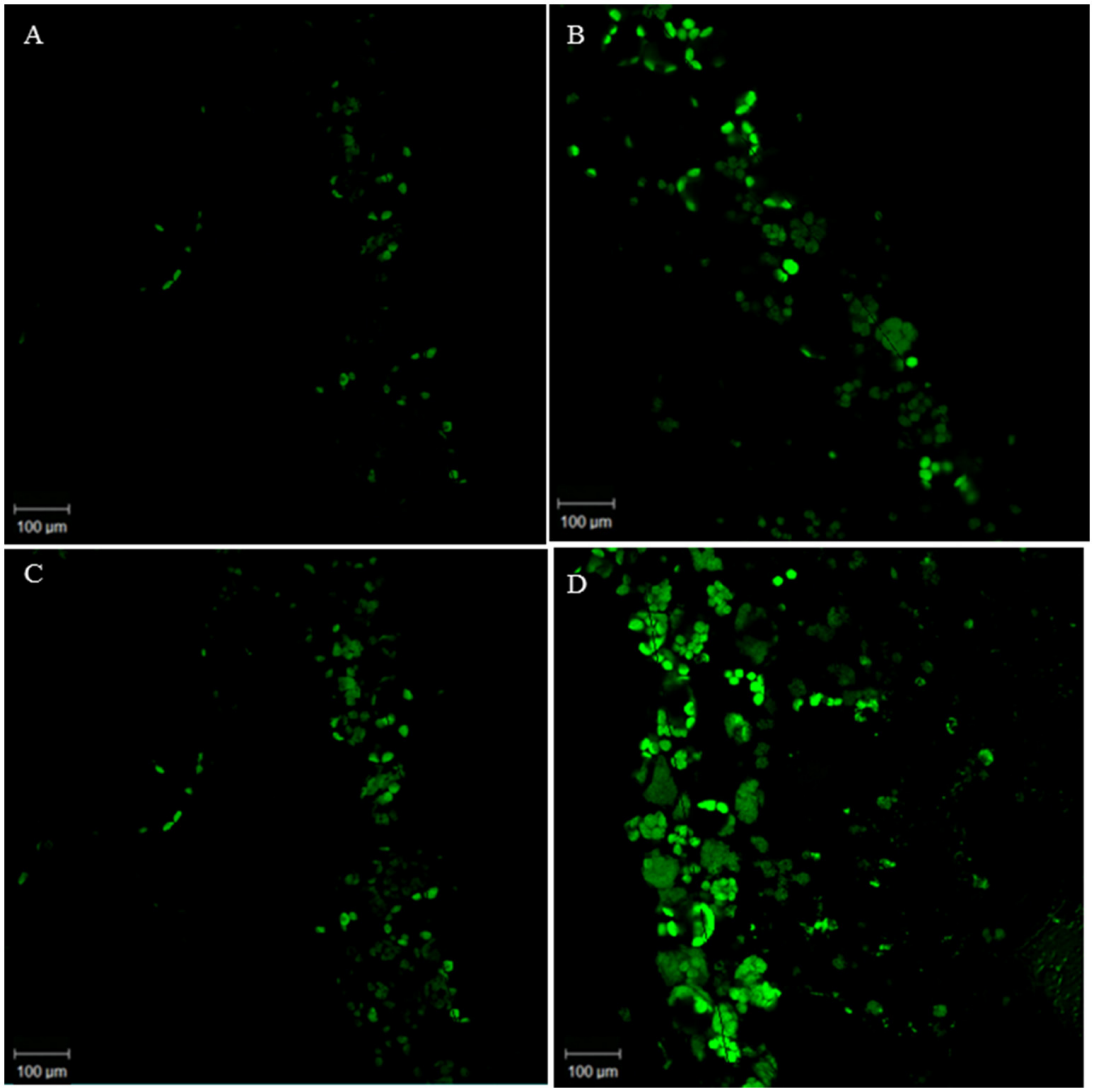
Representative images illustrating the confocal laser immunofluorescent detection of NO in *Pistia stratiotes* leaves. NO was detected by its bright green fluorescence after incubation with DAF-2DA. Control plants with DAF-2DA **(A)**; Plants treated with SNP and DAF-2DA **(B)**; Plants exposed to arsenic and incubated with DAF-2DA **(C)**; Plants exposed to arsenic + SNP and incubated with DAF-2DA **(D)**.

## Conflict of interest statement

The authors declare that the research was conducted in the absence of any commercial or financial relationships that could be construed as a potential conflict of interest.

